# Supplementary material for: Blood Metabolite Profiling of Antarctic Expedition Members: An 1H NMR Spectroscopy-Based Study
Source: Int J Mol Sci. 2023 May 8;24(9):8459. doi: 10.3390/ijms24098459 (PMC10179003; doi:10.3390/ijms24098459)
Supplement: Supplementary file 1 [file ijms-24-08459-s001.zip › ijms-2341183-supplementary.pdf]

Supplementary Materials

Blood Metabolite Profiling of Antarctic Expedition Members: An ^{1}H NMR Spectroscopy-Based Study

Laura Del Coco [1,†], Marco Greco [1,†], Alessandra Inguscio [1], Anas Munir [1,2], Antonio Danieli [1], Luca Cossa [1], Debora Musarò [1], Maria Rosaria Coscia [3], Francesco Paolo Fanizzi [1,*] and Michele Maffia [1,*]

1. Department of Biological and Environmental Science and Technology, University of Salento, Via Lecce—Monteroni, 73100 Lecce, Italy; laura.delcoco@unisalento.it (L.D.C.); marco.greco@unisalento.it (M.G.); ale.inguscio@unisalento.it (A.I.); anas.munir@studenti.unisalento.it (A.M.); antonio.danieli@unisalento.it (A.D.); lucagiulio.cossa@unisalento.it (L.C.); debora.musaro@unisalento.it (D.M.)
2. Department of Mathematics and Physics "E. De Giorgi", University of Salento, Via Lecce—Arnesano, 73100 Lecce, Italy
3. Institute of Biochemistry and Cell Biology, National Research Council of Italy, Via P. Castellino 111, 80131 Naples, Italy; mariarosaria.coscia@ibbc.cnr.it

* Correspondence: fp.fanizzi@unisalento.it (F.P.F.); michele.maffia@unisalento.it (M.M.); Tel.: +0039-0832-299265 (F.P.F.); +0039-0832-298670 (M.M.)

† These authors contributed equally to this work.

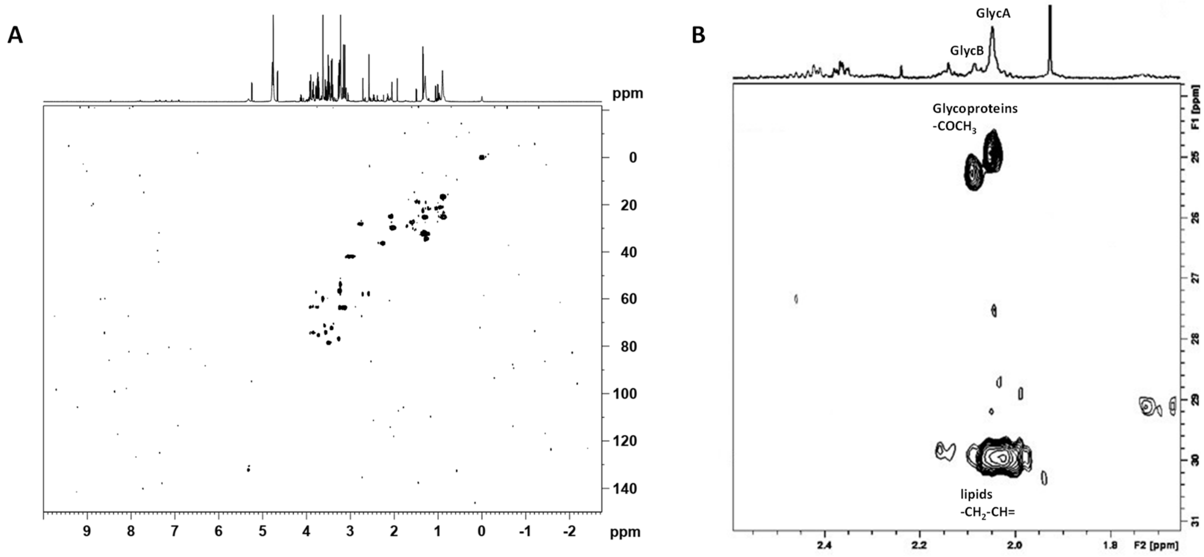

Figure S1. A) 600MHz ^{1}H-^{13}C HSQC spectrum of human serum and **B)** relative expansion in the region 2.5-1.5 ppm (for ^{1}H) and 31-20 ppm (for ^{13}C); -$COCH_3$ acetyl groups of glycoproteins, GlycA and GlycB [19].

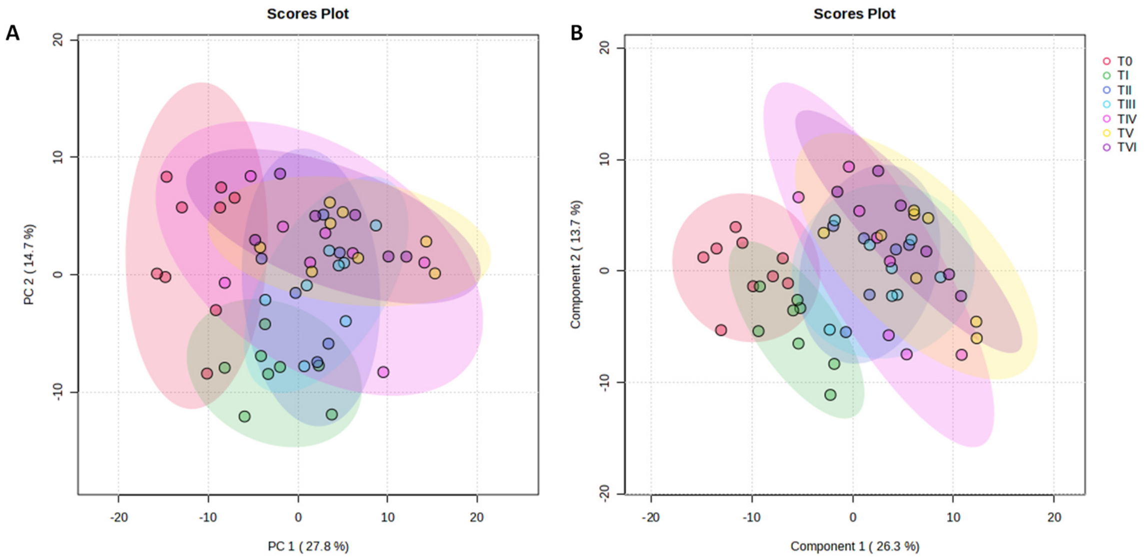

Figure S1. A) PCA and **(B)** PLS-DA score plots obtained from the explorative (PCA model) and supervised (PLS-DA model) analyses performed on the whole spectral data. From statistical analyses, relatively higher levels of glucose, glutamate and N-acetyl glycoproteins (GlycA) and relative low content of lipids and glutamine were found in samples collected at time "T0", with respect to the other serum samples (PCA = principal component analysis; PLS-DA = partial least squares discriminant analysis initial sampling session (T0) and subsequent samplings after 7 (TII), 14 (TIII), 90 (TIV), 180 (TV), and 270 (TVI) days from the arrival.

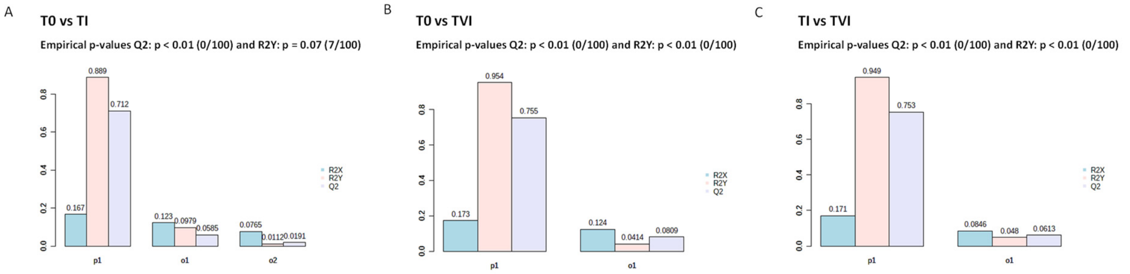

Figure S2. The overview of the OPLS-DA model performance for the selected groups and the permutation test. R^2X and R^2Y refer to the goodness and represent the model interpretation rate. Q^2 refers to the predicted variation (quality of prediction). R^2Y and Q^2 closer to 1 indicate the more stable and reliable model.

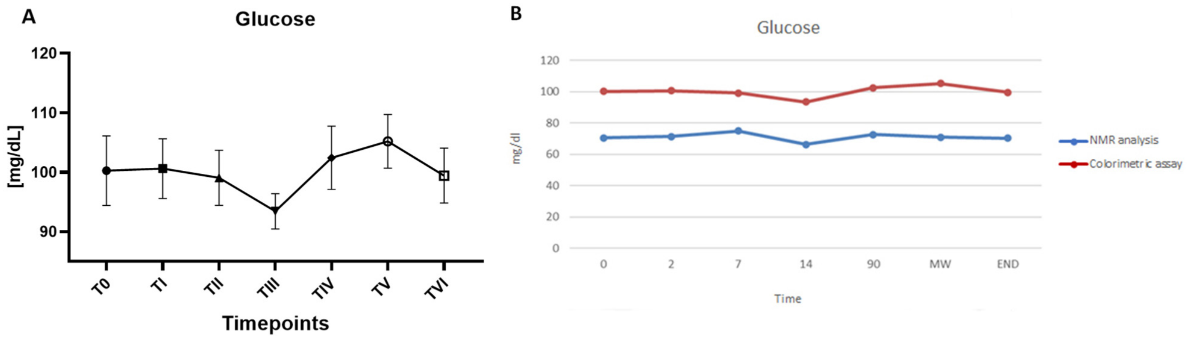

Figure S3. A) Colorimetric assay for glucose circulating levels of subjects in correspondence of the different timepoints. Values are represented as mean ± SEM. Error bar indicates the interindividual fluctuation in the measured concentrations; **B)** Bruker ERETIC measurements used to directly measure the concentration of blood glucose. A good correlation has been obtained considering the concentration (mg/dL) calculated from the NMR spectra (^{1}H NMR signal at 5.25 ppm) and the colorimetric assay.

Table S1. Representative ^{1}H and ^{13}C NMR assignments of metabolites identified in human serum samples at 600 MHz (bs: broad signal; d: doublet; s: singlet; q: quartet; m: multiplet).

**Metabolite**	**δ_H ppm (multiplicity, assignment)/ δ_C ppm**
Acetate	1.92 (s, CH_3)/25.98
Acetoacetate	2.24 (s, CH_3)
Alanine	1.47(d, CH_3)/18.4
Choline	3.20 (s, $N(CH_3)_3$/56.71
Citrate	2.54(d, half CH_2); 2.68(d, half CH_2)
Creatine/phosphocreatine	3.04(s, CH_3)/42.19; 3.96(s, CH_2)
Formate	8.46 (s, CH)
Glycerol	3.58(m, CH_2), 65.4; 3.67 (m, CH_2), 65.4
Glycine	3.57 (s, CH_2)/43.2
Glucose	3.25 (m, CH)/76.96; 3.42(m, CH)/72.5; 3.48(m, CH)/78.6; 3.55(m, CH)/74.27; 3.72(m, CH)/63.3; 3.85(m, CH)/63.4; 3.91/63.53; (m, CH_2); 4.64(d, CH); 5.24(d, CH)/94.88
Glutamate	2.08(m, half CH_2)/29.84; 2.13(m, half CH_2)/29.86; 2.36 (m, CH_2)/36.4; 3.78 (m, CH)/57.21
Glutamine	2.15(m, half CH_2)/29.88; 2.42(m, half CH_2)/36.48
3-Hydroxybutyrate	1.20(d, CH_3)/21.61
Histidine	7.07 (s, CH); 7.79 (s, CH)
Isoleucine	0.94(t, CH_3)13.69; 1.02 (d, CH_3)
Lactate	1.33(d, CH_3)/22.9; 4.12 (q, CH)/71.4
Leucine	0.98(t, CH_3)/24.8; 1.73 (m, CH, CH_2)/42.72
Lipids, LDL	0.87(bs, CH_3)/16.74; 1.29(bs, CH_{2n})/34.7; 1.57(m, $C\mathbf{H}_2CH_2COOC$)/27.53; 2.01(m, $C\mathbf{H_2}CH=$)/29.9; 5.32(m, $C\mathbf{H}=C\mathbf{H}$)/132.43
Lipids, VLDL	0.90(bs, CH_3)/25.33; 1.32(bs, CH_{2n})/32.35; 1.57(m, CH_2CH_2COOC)/27.53; 2.01(m, $C\mathbf{H_2}CH=$)/29.9; 5.32(m, $C\mathbf{H}=C\mathbf{H}$)/132.43
N-acetyl glycoproteins	2.05-2.07 (bs, $COC\mathbf{H_3}$)/29.92
Phenylalanine	7.34(d, CH ring); 7.39(m, CH ring); 7.43(m, CH ring)
Tyrosine	6.90 (d, CH ring); 7.20 (d, CH ring)
Unidentified	2.82 (s, CH_3)/32.53
Valine	1.00(d, CH_3)/19.7; 1.05 (d, CH_3)/20.7; 2.28 (m, CH)/31.9

Table S2. The significant differences of the mean values, for all the time-points, from T0 to subsequent samplings after 7 (TII), 14 (TIII), 90 (TIV), 180 (TV), and 270 (TVI) days after arrival were obtained by one-way ANOVA followed by Tukey's honestly significant differences (HSD) post hoc test and repeated one-way ANOVA (Core Team (2021). R: A language and environment for statistical computing. R Foundation for Statistical Computing, Vienna, Austria.URL https://www.R-project.org/).

**Metabolite assignment**	**Binned NMR peak (ppm)**	***p*-value**	**Post hoc tests**
**Glutamate**	2.37	$1.61*10^{-10}$	t1-t0; t2-t0; t3-t0; t4-t0; t5-t0; t6-t0
**Glutamine**	2.47	$1.36*10^{-9}$	t1-t0; t2-t0; t3-t0; t4-t0; t5-t0; t6-t0; t4-t1; t5-t1; t6-t1; t4-t2; t6-t2
**Lipids**	0.89	$7.28 *10^{-4}$	t5-t0; t6-t0; t5-t1
***N*-acetyl glycoproteins**	2.05	$1.09*10^{-8}$	t2-t0; t3-t0; t4-t0; t5-t0; t6-t0; t3-t1; t4-t1; t5-t1; t6-t1

**Lipids (bin: 0.89)**
**Univariate Type II Repeated-Measures ANOVA**
Sum Sq num Df Error SS den Df F value Pr(>F)
(Intercept) 0.072297 1 0.0050777 44 626.4811 < 2.2e-16 ***
subject 0.002688 7 0.0050777 44 3.3275 0.006252 **
TIME 0.000000 6 0.0000000 264 NaN NaN
subject:TIME 0.000000 42 0.0000000 264 NaN NaN

**Glutamate (bin: 2.37)**
**Univariate Type II Repeated-Measures ANOVA**
Sum Sq num Df Error SS den Df F value Pr(>F)
(Intercept) 0.011432 1 0.00070641 44 712.0919 <2e-16 ***
subject 0.000252 7 0.00070641 44 2.2423 0.0485 *
TIME 0.000000 6 0.00000000 264 NaN NaN
subject:TIME 0.000000 42 0.00000000 264 NaN NaN

**Glutamine (bin: 2.47)**
**Univariate Type II Repeated-Measures ANOVA**
Sum Sq num Df Error SS den Df F value Pr(>F)
(Intercept) 0.0081472 1 0.0005836 44 614.2580 < 2e-16 ***
subject 0.0002288 7 0.0005836 44 2.4641 0.03184 *
TIME 0.0000000 6 0.0000000 264 NaN NaN
subject:TIME 0.0000000 42 0.0000000 264 NaN NaN

***N*-acetyl-glycoproteins (bin: 2.05)**
**Univariate Type II Repeated-Measures ANOVA**
Sum Sq num Df Error SS den Df F value Pr(>F)
(Intercept) 0.182367 1 0.0030815 44 2603.9466 <2e-16 ***
subject 0.000644 7 0.0030815 44 1.3131 0.2668
TIME 0.000000 6 0.0000000 264 NaN NaN
subject:TIME 0.000000 42 0.0000000 264 NaN NaN

Signif. codes: 0 '***' 0.001 '**' 0.01 '*' 0.05 '.' 0.1 ' ' 1

Table S3. Results from Pathway Analysis with MetPA for serum samples T0 vs TI (Total number of compounds involved in each pathway and metabolites matched from the uploaded data; *p* is the original *p*-value calculated from the enrichment analysis; the impact is the pathway impact value calculated from pathway topology analysis).

ID	Pathway name	Total	Hits	*p*-value	-log(*p*)	Holm *p*	FDR	Impact
**A**	**D-glutamine and D-glutamate metabolism**	6	2	0.0001758	6.755	0.028128	0.0004688	**0.50**
**B**	**Alanine, aspartate and glutamate metabolism**	28	2	0.0001758	6.755	0.028128	0.0004688	**0.3109**
**C**	**Arginine biosynthesis**	14	2	0.0001758	6.755	0.028128	0.0004688	**0.11675**

Table S4. Results from Pathway Analysis with MetPA for serum samples T0 vs TVI (Total number of compounds involved in each pathway and metabolites matched from the uploaded data; *p* is the original *p*-value calculated from the enrichment analysis; the impact is the pathway impact value calculated from pathway topology analysis).

ID	Pathway name	Total	Hits	*p*-value	-log(*p*)	Holm *p*	FDR	Impact
**A**	**D-glutamine and D-glutamate metabolism**	6	1	2.06E-04	76.851	2.06E-03	2.06E-04	**0.5**
**B**	**Alanine, aspartate and glutamate metabolism**	28	1	2.06E-04	76.851	2.06E-03	2.06E-04	**0.19712**
**C**	**Arginine biosynthesis**	14	1	2.06E-04	76.851	2.06E-03	2.06E-04	**0.11675**

Table S5. Results from Pathway Analysis with MetPA for serum samples TI vs TVI (Total number of compounds involved in each pathway and metabolites matched from the uploaded data; *p* is the original *p*-value calculated from the enrichment analysis; the impact is the pathway impact value calculated from pathway topology analysis).

ID	Pathway name	Total	Hits	*p*-value	-log(*p*)	Holm *p*	FDR	Impact
**A**	**Phenylalanine, tyrosine, and tryptophan biosynthesis**	4	2	0.0066599	61.765	0.053279	0.001776	**1.0**
**B**	**Phenylalanine metabolism**	10	2	0.0066599	61.765	0.053279	0.001776	**0.35714**

Table S6. List of collected samples at different test sampling time (days).

**Blood test sampling time (days)**							
**Sample ID**	**T0**	**TI**	**TII**	**TIII**	**TIV**	**TV**	**TVI**
A	0	2	7	14	90	MW	END
B	0	2	7	14	90	MW	END
C	0	2	7	14	90	MW	END
D	0	2	7	14	90	MW	END
F	0	2	7	14	90	MW	END
G	0	2	7	14	90	MW	END
H	0	2	7	14	90	MW	END
K	0	2	7	14	90	MW	END
